# Body position to optimize mechanics in ARDS: to which degree does the angle matter?

**DOI:** 10.1186/s40635-023-00560-0

**Published:** 2023-11-15

**Authors:** Jantine Wisse, Annemijn H. Jonkman

**Affiliations:** Department of Intensive Care Adults, Erasmus Medical Center, Rotterdam, The Netherlands

The inherent heterogeneity of the acute respiratory distress syndrome (ARDS) provides a strong rationale for applying mechanical ventilation strategies tailored to the individual patient’s characteristics. Such precision medicine approach is increasingly recognized [1]. It also requires standardized assessment of respiratory mechanics to systematically evaluate the patient’s response to treatment or to adjustments in ventilator settings [2]. One important but maybe overlooked modifier of this response is the patient’s body position. Changes in body position can quickly alter respiratory mechanics by adjusting airway resistance and lung and chest wall compliance [3]. Recently, the supine-flat compared to semi-recumbent position was found to improve CO_2_ clearance in COVID-19 ARDS patients after brief position changes [4]. The underlying physiological mechanisms but also the longer effects of trunk inclination on (regional) mechanics and ventilatory efficiency are, however, incompletely understood.

In this Journal’s issue, Benites et al. [5] investigated in 22 patients with (mostly COVID-19) ARDS the effect of trunk inclination from semi-recumbent (45°) to a supine-flat (10°) and back to semi-recumbent position on physiological dead space and regional ventilation distribution. Steps were applied for 60 min each and sophisticated respiratory monitoring using volumetric capnography and electrical impedance tomography was performed. Main findings were that trunk inclination from 45° to 10° improved Bohr’s dead space and decreased PaCO_2_, and resulted in a redistribution of ventilation to the dorsal lung region despite no changes in global lung inhomogeneity and tidal volume.

Should we now put all our ventilated ARDS patients in flat-supine position to optimize their mechanics? We are not sure yet. Benites’s study revealed a large variability in the response to trunk inclination, which was likely partly driven by the heterogeneity in ARDS severity of patients enrolled (study enrolment criteria ranged from mild to severe ARDS). For instance, despite no significant group-level changes in global and regional end-expiratory lung impedance (EELI), individual patients could show either increases or decreases in ventral and/or dorsal EELI. Increased EELI indicates improved lung aeration. Potentially, the position-dependent effect on lung aeration may aid in identifying those patients, whose respiratory mechanics could benefit from a body position change. From a physiological perspective, the amount of recruitable lung tissue could influence the response to trunk inclination [3]. Shifting from supine-flat to semi-recumbent position causes the diaphragm to move downwards, lowering the risks of lung compression by the weight of the mediastinum, heart and abdomen. In the healthy lung, this generally increases transpulmonary pressure and lung volume (and thus compliance). With severe ARDS and non-recruitable lungs, a paradoxical response may occur as an increased transpulmonary pressure in a more upright position would result in no or minimal recruitment and mostly overdistention [3].

Especially, since the pandemic, there has been increasing interest in applying very simple maneuvers to modify respiratory mechanics, including changes in body position, and chest wall or abdominal loading. Physiological research is needed to determine the response and therapeutic potential of such maneuvers and the elegant study of Benites et al. [5] provides important new knowledge. At the same time, the authors highlight the need for reporting of body position when systematically assessing respiratory mechanics in ARDS. Their results again underline the importance of personalized approaches in mechanical ventilation.

## Data Availability

Not applicable.
